# Special Issue “Latest Review Papers in Molecular Genetics and Genomics 2023”

**DOI:** 10.3390/ijms241814171

**Published:** 2023-09-16

**Authors:** Apostolos Zaravinos

**Affiliations:** 1 Department of Life Sciences, School of Sciences, European University Cyprus, 2404 Nicosia, Cyprus; a.zaravinos@euc.ac.cy; 2 Cancer Genetics, Genomics and Systems Biology Laboratory, Basic and Translational Cancer Research Center (BTCRC), 2404 Nicosia, Cyprus

In the rapidly evolving landscape of molecular genetics and genomics, this Special Issue brings together a collection of insightful review articles that delve into the forefront of scientific exploration. 

The Special Issue titled “Latest Advances in Molecular Genetics and Genomics (2023)” of the International Journal of Molecular Sciences includes nine reviews underscoring the remarkable strides made in our understanding of fundamental biological processes and their implications for human health and disease. Spanning diverse topics, the nine articles presented herein illuminate various facets of molecular genetics and genomics, shedding light on the intricate interplay of genetic information, cellular pathways, and disease mechanisms.

A pivotal theme that emerges from these articles is the burgeoning role of non-coding RNAs in shaping gene expression and cellular function. The intricate regulatory networks orchestrated by microRNAs (miRNAs) and circular RNAs (circRNAs) have garnered significant attention as they control gene expression at the transcriptional and post-transcriptional levels and, thus, play a critical role in tumorigenesis and cancer progression. The work by Kim et al. [1] reveals the intricate circular RNA–microRNA axis in cancer, where these non-coding RNAs engage in a complex interplay, influencing tumorigenesis and progression across multiple cancer types. The authors focus on the biological regulation of the correlative axis among circular RNAs, miRNAs, and their target genes in various cancer types and suggest the biological importance of miRNAs derived from transposable elements (MDTEs) interacting with oncogenic or tumor-suppressive circRNAs in tumor progression [1].

Similarly, the study by Pierzynowska et al. [2] underscores the multifaceted involvement of the oxytocin receptor (OXTR) and its ligand in various diseases, highlighting the delicate balance between behavior, molecular signaling, and pathogenesis. The authors summarize the involvement of OXTR dysfunctions and polymorphisms in the development of different diseases, including cancer, cardiac disorders, osteoporosis, and obesity. Their analysis suggests that changes in OXTR expression and OXTR abundance and activity are not specific to individual diseases but rather influence processes that might modulate the course of various disorders. The authors also propose a possible explanation of the discrepancies in the published results of the effects of the OXTR gene polymorphisms and methylation on different diseases [2].

Advances in technology have also enabled comprehensive exploration of genetic alterations and their clinical implications. The investigation by Aveta et al. [3] comprehensively surveys urinary microRNAs (umiRNAs) as potential biomarkers for urological cancers. This systematic review offers insights into the diagnostic and prognostic potential of umiRNAs, illuminating new avenues for non-invasive cancer detection and monitoring. Their systematic review suggests that umiRNAs could play an important role in the diagnosis, prognosis, and therapy of urological cancers [3].

Additionally, the study by Moeckel et al. [4] delves into the promise of tumor mutation burden (TMB) as a predictive biomarker for immune checkpoint inhibitor response. The authors discuss the cellular composition in the tumor microenvironment (TME) and the variability in gene mutations across different tumors. They also describe the association between mutations and the response of the immune system, as well as how TMB can be used as a potential biomarker to predict such a response to immune checkpoint inhibitors (ICI). The intricacies of TMB in different cancer types are meticulously examined, emphasizing the need for personalized approaches to harness its predictive power effectively and overcome immunoresistance to ICI therapies. The authors further highlight new trends in the field, such as liquid biopsies, next-generation sequencing, chimeric antigen receptor T-cell therapy, and personalized tumor vaccines [4].

The integration of genomics and molecular mechanisms is underscored by investigations into gene architecture and regulation. Kyrchanova et al. [5] provide a detailed exploration of enhancer–promoter interactions in Drosophila, revealing insights into the orchestration of long-range gene regulation. Their work describes the current progress in the interactions at three well-studied key regulatory loci in Drosophila [5]. Specifically, they describe the different models that have been used to decipher the distance interactions between regulatory elements, as well as the current models of communication between enhancers and promoters. In addition, the authors discuss the role of the interacting insulators from an autonomous regulatory domain of the eve gene in Drosophila. They further explain how insulators and tethering elements provide independent regulation of genes in the antennapedia gene complex, as well as how boundaries organize the enhancer–promoter interactions in the Abd-B gene of the bithorax complex in Drosophila [5].

Meanwhile, Franzago et al. [6] shed light on the emerging field of chrono-nutrition (i.e., the study of the impact of the timing of eating by matching elements from nutritional research with chrono-biology), where the intersection of circadian rhythms and personalized nutrition holds promise for mitigating chronic diseases through tailored dietary strategies. Overall, this review provides an overview of the current evidence on the interactions between the circadian system and nutrition, highlighting how this link could, in turn, influence the epigenome and microbiome. In addition, the authors suggest possible nutritional strategies to manage circadian-aligned feeding [6].

Other articles in this Special Issue delve into the significance of germline and somatic alterations in key genes like MRE11, RAD50, and NBN and offer mechanistic insights into IQSEC2 disease and the use of *Schmidtea mediterranea* as a model organism for studying human motile ciliopathies. 

Specifically, Otahalova et al. [7] outline the structural characteristics of the MRE11, RAD50, and NBN proteins and the assembly and functions of the MRN complex from the perspective of clinical interpretation of germline and somatic alterations in the MRE11, RAD50, and NBN genes. They describe the structure of the MRN Complex and its function in double-strand break (DSB) repair and analyze the different germline alterations of the MRN complex genes in autosomal recessive syndromes, as well as the association between heterozygous germline alterations in these genes and cancer predisposition. Their review finally explores the role of somatic alterations in the MRN complex genes in different tumors [7].

Furthermore, the review by Levy et al. [8] outlines recent insights into IQSEC2 disease (an X-linked disorder associated with intellectual disability, autism, and epilepsy), including the identification of missense mutations in functional domains through patient DNA sequencing, recapitulation of autistic-like behavior, and seizures in transgenic mouse models. The review highlights the role of IQSEC2 in neurotransmission and neuronal development, the constitutively high levels of Arf6-GTP in knockout mice, and the potential therapeutic benefit of heat treatment for the IQSEC2 A350V mutation [8].

Lastly, Rabiasz et al. [9] discuss the ultrastructure of motile cilia and review the use of this simple and accessible planarian model for studying the genetics of primary ciliary dyskinesia (PCD) and other cilia-related diseases.

As we reflect on these diverse contributions in the Special Issue “Latest Advances in Molecular Genetics and Genomics (2023)”, a common thread emerges: the intricate link between genetics, molecular mechanisms, and disease. These review papers collectively underscore the remarkable strides we have taken in unraveling the molecular complexities of different diseases while also highlighting the tantalizing prospects that lie ahead. 

Overall, the studies in this Special Issue highlight the latest developments in molecular genetics and genomics and improve our current understanding of key mechanisms involved in this field. These findings not only showcase the accomplishments of the past but also pave the way for exciting avenues of further investigation in the field of molecular genetics and genomics.

In closing, I would like to extend my deepest gratitude to the authors, reviewers, and Editorial team for their invaluable contributions to this Special Issue. I hope that these articles will not only enhance our understanding of molecular genetics and genomics but also inspire further inquiry and innovation in this dynamic and ever-evolving field.

## Renference
